# Tirzepatide, the Newest Medication for Type 2 Diabetes: A Review of the Literature and Implications for Clinical Practice

**DOI:** 10.1177/10600280231224039

**Published:** 2024-01-21

**Authors:** Sarita Jacob, George I. Varughese

Bradley et al highlight the demonstrable benefits of Tirzepatide, a dual incretin mimetic in terms of greater HbA1c lowering and weight reduction and no further increase in risk of retinopathy compared to GLP-1 receptor agonists (GLP-1RA).^1^ In this context, it is important to be cautious and remember the superior benefits of Tirzepatide in terms of glycemic control in conjunction with weight reduction and the magnitude of improvement in HbA1c compared with GLP-1RA, with the risk of early and transient worsening of pre-existing diabetic retinopathy (DR).^2^ This specific paradox should not be underestimated by clinicians and allied health care prescribers of newer medications, especially GLP-1RA for type 2 diabetes mellitus (T2DM).

The majority of evidence supports an association of large and rapid reductions in blood-glucose levels, higher HbA1c at the outset, and pre-existing DR.^2^ Despite a general awareness of early worsening within the diabetes fraternity, the exact mechanism of action is not conclusive. With the first GLP-1RA Exenatide, a retrospective analysis of patients receiving treatment twice daily for longer than 6 months showed that DR had progressed in 30% of patients and progression of DR was associated with greater reductions in HbA1c; there was no comparator.^3^ In the same group of patients, DR had improved or remained stable after follow-up, suggesting that early worsening did not progress.^4^ Importantly, this follow-up study applied robust methods for evaluating DR prospectively,^3,4^ whereas the majority of the DR findings relating to GLP-1RA were based on standard adverse event reporting and not retinal imaging.

Semaglutide is the latest GLP-1RA used both orally as a daily treatment or subcutaneously once a week for the management of T2DM,^5-7^ with impressive results. However, during the SUSTAIN clinical trial program, the safety issue concerning the progression and worsening of DR once again emerged and was indeed very conspicuous.^5-8^ Cardiovascular outcome trials provide the longest available randomized, placebo-controlled follow-up for GLP-1RAs^9,10^; however, none of these trials were designed or powered to provide robust estimates of GLP-1RA effects on DR.

Dual incretin mimetics, gastric inhibitory polypeptide (GIP) and GLP-1RA agonist, Tirzepatide the first in class drug has demonstrated more significant improvements in weight and optimization of glucose control in T2DM.^1^ The impetus of the additive effect of GIP and GLP-1RA dual therapy in T2DM and on glucose control and weight reduction, respectively, is very relevant in this context. Of note, this could potentially pose further synergistic repercussions on the early progression of DR, and it is prudent to watch out for this clinical paradox in this at-risk cohort of patients with diabetes.

A joint collaborative working partnership with the diabetic eye screening programs and ophthalmologists would be essential in these challenging circumstances,^10^ and closer monitoring for changes in DR status becomes even more important.^5,6^ The most recent diabetic eye screening report needs to be reviewed prior to initiation of these drugs and is certainly recommended to adopt and adapt a careful watch and to observe this well-recognized, but less commonly perceived phenomenon with swift improvement in glucose levels and dual incretin mimetics.


Sarita Jacob, MRCS Ed, MRCOphth, FAcadMed *University Hospitals Birmingham NHS Foundation Trust, Birmingham, UK*
George I. Varughese, FRCP (Edin), FRCPI, FRCP
*University Hospitals of North Midlands NHS Trust, Stoke-on-Trent, UK*

